# Effects of drought on leaf carbon source and growth of European beech are modulated by soil type

**DOI:** 10.1038/srep42462

**Published:** 2017-02-14

**Authors:** Jian-Feng Liu, Matthias Arend, Wen-Juan Yang, Marcus Schaub, Yan-Yan Ni, Arthur Gessler, Ze-Ping Jiang, Andreas Rigling, Mai-He Li

**Affiliations:** 1 State Key Laboratory of Tree Genetics and Breeding, Key Laboratory of Tree Breeding and Cultivation of State Forestry Administration, Research Institute of Forestry, Chinese Academy of Forestry, Beijing, China; 2 Swiss Federal Research Institute WSL, CH-8903 Birmensdorf, Switzerland; 3 Institute of Botany, University of Basel, Basel, Switzerland; 4 Berlin-Brandenburg Institute of Advanced Biodiversity Research (BBIB), Berlin, Germany; 5 Institute of Applied Ecology, Chinese Academy of Sciences, Shenyang, China

## Abstract

Drought potentially affects carbon balance and growth of trees, but little is known to what extent soil plays a role in the trade-off between carbon gain and growth investment. In the present study, we analyzed leaf non-structural carbohydrates (NSC) as an indicator of the balance of photosynthetic carbon gain and carbon use, as well as growth of European beech (*Fagus sylvatica* L.) saplings, which were grown on two different soil types (calcareous and acidic) in model ecosystems and subjected to a severe summer drought. Our results showed that drought led in general to increased total NSC concentrations and to decreased growth rate, and drought reduced shoot and stem growth of plants in acidic soil rather than in calcareous soil. This result indicated that soil type modulated the carbon trade-off between net leaf carbon gain and carbon investment to growth. In drought-stressed trees, leaf starch concentration and growth correlated negatively whereas soluble sugar:starch ratio and growth correlated positively, which may contribute to a better understanding of growth regulation under drought conditions. Our results emphasize the role of soil in determining the trade-off between the balance of carbon gain and carbon use on the leaf level and growth under stress (e.g. drought).

Drought is expected to become an increasingly important stressor in many ecosystems^1^, not only determining forest species growth and productivity^2^,^3^, but also persistence and distribution patterns of species^4^,^5^. The early life stages of trees are most sensitive and vulnerable to soil water deficit^6^,^7^. Progressive drought may influence patterns of tree seedling and sapling establishment and have lasting effects on the composition, dynamics and carbon balance of forests^8^,^9^. Meanwhile, progressive drought may affect reforestation projects, where thousands of newly planted saplings die within the first few years of planting^10^. Furthermore, extreme drought may lead to forest dieback, potentially converting forests from a net carbon sink into a large carbon source^11^. Thus, understanding how trees in early stage respond to drought is crucial for predicting the fate of forest ecosystems under future climate conditions^12^,^13^.

The plant non-structural carbohydrates (NSC consisting of soluble sugars and starch) formed in leaves during photosynthesis are on the one hand serving as central crossroad in the leaf metabolism^14^ and are used to supply heterotrophic plant organs with carbon and energy via the phloem on the other hand. Starch accumulates in the chloroplasts in plant leaves either being under internal control to suit the environmental conditions^15^ or being induced due to either high carbon assimilation or low carbon export^16^. Drought, imposed as reduced soil water availability and/or atmospheric drought, constrains plant physiology and productivity through the reduction of leaf gas exchange associated with reduced carbon gain, and growth activity associated with carbon investment, which affects the carbon balance in leaves^17^,^18^. Thus levels of leaf NSC reflect the balance between carbon gain and carbon utilization with respect to the entire plant source and sink activity. Moreover, leaf or tissue NSC might also act as short-term buffer during insufficient source activities due to environmental stress^19^. Generally, growth (i.e. cambial activity) is most sensitive to drought, followed by photosynthesis and respiration (see review by McDowell^20^). Up to now, most studies have found either no reduction or even an increase in NSC levels of trees under moderate drought^21^,^22^,^23^,^24^ and it has been speculated that the higher drought sensitivity of the sink activity compared to photosynthesis might be responsible for such transient increased. However, whether this pattern might also be related to source (leaf) carbon storage affecting carbon sink activity (growth) is not clear^22^,^25^,^26^.

Apart from the large number of studies on plant eco-physiological responses to drought, little is known about the effects of soil nutrient status on growth of trees under drought. Among the nutrients needed by plants, nitrogen and phosphorus play vital roles in physiological functioning, and are among the most important limiting nutrients in terrestrial ecosystems^27^,^28^,^29^. Leaf nitrogen and phosphorus concentrations, which are determined by uptake and loss, can reflect the relationship between plant and soil nutrient status^30^. The variations in leaf nitrogen/phosphorus ratio are species-specific, and depend on nutrient conditions to which plants are exposed^31^. Nutrient imbalances in plants may also lead to reduced soil nutrient availability to plant growth^32^,^33^. However, the effects of nutrient status on the plant’s response to drought are less well-known. Up to now, only a few reports suggest that the medium-term drought reduces root nutrient uptake activity^34^ and nutrient availability in soils^35^,^36^, and thus may lead to reduction of nitrogen and phosphorus concentrations in stand biomass^36^. Low nutrient availability may reduce plant water use efficiency and capacity to adapt to drought^37^.

Differences in the physiology of the nutrient uptake system may contribute to species-specific variations in drought tolerance^38^, and soil properties which determine nutrient availability and soil water relations may also play a role in plants’ drought tolerance. For instance, calcareous soils often have a much lower water-holding capacity than acidic soils^39^. As a consequence, trees growing on calcareous soil have to cope with more intense drought conditions compared to trees growing on acidic soil but plants on the former soil often show a higher drought tolerance than those growing on acidic soils^40^,^41^. Thiel *et al*.^42^ found that the growth performances of *Fagus sylvatica* L. under drought differed between soil types (sand and loam), where a higher growth reduction was found in the sandy substrate. However, whether the soil type modifies the carbon source-sink relationship and thus plant growth is still unknown.

European beech (*Fagus sylvatica* L.) is an ecologically dominant tree species in Central Europe occupying a wide range of mesic soils with contrasting pH and carbonate content^43^. It is commonly considered as a drought-sensitive species^44^,^45^, especially during early stages of establishment^46^, but it has also been reported that seedlings recover quickly from severe drought episodes and provenances may differ in their drought and post-drought response^47^,^48^,^49^,^50^,^51^. To date, the influence of soil type has been rarely considered as an additional factor in drought experiments, although it can interact with other environmental constraints^52^,^53^. The present study was undertaken in the framework of the interdisciplinary experiment “BuKlim: beech in a changing climate”^53^ investigating drought and post-drought responses of European beech provenances on different soil types. By analyzing levels of NSCs, nitrogen, and phosphorus in relation to growth, we aimed at testing: (1) how severe drought influences the relationship between leaf carbon assimilation and NSC export associated with tree growth (i.e. leaf NSC balance), and (2) whether soil types affect this relationship.

## Results

### Non-structural carbohydrates (NSCs)

**Figure 1:**
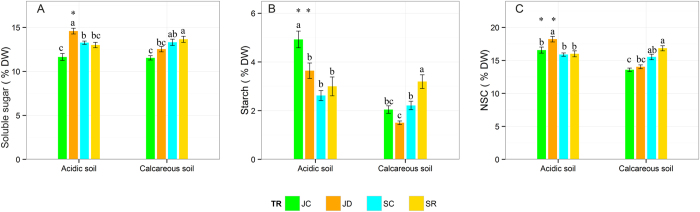
Mean values (±SE) of concentrations (% DW) for soluble sugars (**A**), starch (**B**) and total NSC (**C**) from *Fagus sylvatica* L. leaves grown on acidic and calcareous soils and exposed to drought and re-watering treatments. Asterisks indicate significant differences between acidic and calcareous soils for a certain treatment. Different lowercase letters indicate significant differences between treatments within one soil type (p < 0.05). (JC: control in July; JD: drought in July; SC: control in September; SR: drought (re-watering) in September).

Highly significant effects were found for treatment and the interaction between treatment and sampling period on leaf soluble sugar concentration (% DW) (p < 0.01, Table 1). Drought increased leaf soluble sugar concentration on acidic soil by +25.2% relative to the control (p < 0.05), but no increase was observed on calcareous soil (Fig. 1A). After re-watering (50d), soluble sugar concentration fully recovered to the control level on both soil types.

Leaf starch concentration (% DW) was significantly affected by soil type and the interactions between soil type and sampling period and between treatment and sampling period (p < 0.001, Table 1). Drought decreased the starch concentration by 26% in plants on acidic soil (p < 0.05), but no significant decrease was observed on calcareous soil (Fig. 1B). After re-watering, leaf starch concentration on acidic soil has slightly increased in previously drought-exposed re-watered plants (3.00% DW) compared to controls (2.62% DW) (p > 0.05), but on the calcareous soil, this increase was much more pronounced, rising from 2.22% DW to 3.20% DW (p < 0.05).

There was a significant effect of treatment, soil type and interaction between soil type and sampling period on total NSC concentrations (% DW) (p = 0.01, Table 1). The pattern of total NSC concentrations affected by the treatments was similar to the one of soluble sugar on both soils (Fig. 1C). Drought increased NSC on acidic soil (p < 0.05), but not so on calcareous soil. For the latter, total NSC concentrations in the control (September) and re-watering treatment was higher than the total NSC concentrations in the drought treatment before re-watering (p < 0.05). In summary, concentrations of soluble sugars, starch, and NSC on acidic soil were higher than on calcareous soil in both, the control or drought treatment in July (p < 0.05). After re-watering, no soil-related differences were observed (Fig. 1C).

### Leaf nutrients

**Figure 2:**
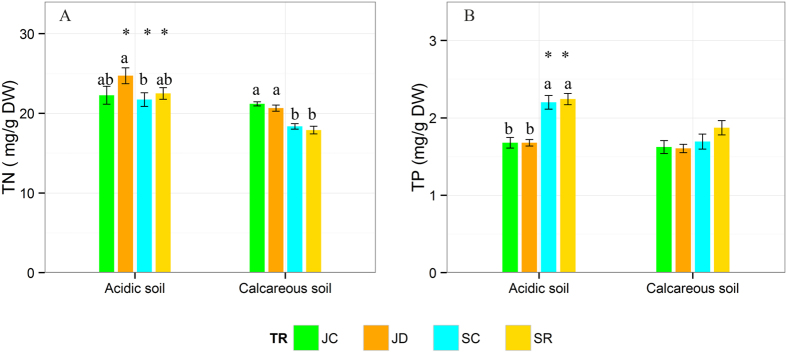
Mean values (±SE) of concentrations (mg/g DW) for nitrogen (**A**), phosphorous (**B**) from *Fagus sylvatica* L. leaves grown on acidic and calcareous soils and exposed to drought and re-watering treatments. Asterisks indicate significant differences between acidic or calcareous soil for a certain treatment. Lowercase letters indicate significant differences between treatments within one soil type (p < 0.05). (JC: control in July; JD: drought in July; SC: control in September; SR: drought (re-watering) in September).

Leaf nitrogen and phosphorus concentrations significantly differed between the two soil types (p < 0.001, Table 1). Furthermore, the interaction between soil type and treatment significantly affected leaf nitrogen concentration (p < 0.05), while the interaction between soil type and sampling period significantly affected leaf phosphorus concentration (p = 0.001). Both, drought and re-watering had no effects on the leaf nitrogen and phosphorous concentrations over the two soil types compared to the corresponding control (Fig. 2A,B). Leaf nitrogen concentration on acidic soil was higher than on calcareous soil across two sampling periods, while only during the latter period (September) this was the case for leaf phosphorus concentration (p < 0.05) (Fig. 2A,B).

### Growth

**Figure 3:**
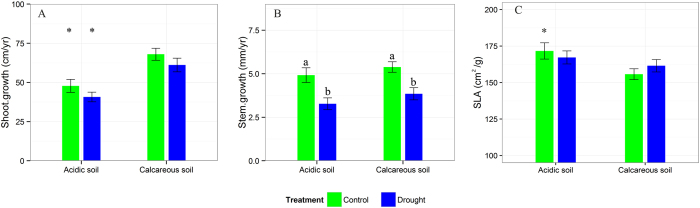
Mean values (±SE) for shoot growth (cm/yr) (**A**), stem diameter growth (mm/yr) (**B**) and SLA (cm^2^/g) (**C**) from *Fagus sylvatica* L. saplings grown on acidic and calcareous soils and exposed to drought and re-watering treatments. Asterisks indicate significant differences between acidic and calcareous soil for a certain treatment. Different lowercase letters denote significant differences between treatments within a soil type (p < 0.05).

The two factors, treatment (control *vs*. drought) and soil (acidic *vs*. calcareous), had distinct effects on SLA, annual shoot, and stem increment but no interactive effects on them (Table 2). Shoot increment and SLA were significantly affected by soil (p < 0.05), while stem increment by treatment (p < 0.001). Overall, saplings grown on calcareous soil had higher annual shoot increment than on acidic soil (p < 0.05), with 68.00 ± 3.80 cm in control and 61.13 ± 4.35 cm in the drought treatment compared to 47.80 ± 4.13 cm in control and 40.73 ± 3.08 cm in the drought treatment on acidic soil (Fig. 3A). The drought treatment reduced the stem increment by 33.55% on acidic soil (p < 0.05), with 4.92 ± 0.42 mm for control and 3.27 ± 0.33 mm for drought, and by 28.6% on calcareous soil (p < 0.05), with 5.39 ± 0.30 mm for control and 3.85 ± 0.35 mm for drought (Fig. 3B). Opposite to shoot increment, the saplings had higher SLA on acidic soil than on calcareous soil. For example, SLA was 171.67 ± 5.55 cm^2^/g on acidic soil and 155.74 ± 3.68 cm^2^/g on calcareous soil for plants in the control treatment (Fig. 3C).

### Correlation between resources and growth

According to Konôpka *et al*.^54^ and Michelot *et al*.^55^, height and diameter increments of European beech almost ceased in July. We therefore investigated the correlation between resources (including leaf soluble sugar, starch, NSC, ratio of soluble sugar to starch, nitrogen and phosphorus concentrations) in July and the annual growth status (shoot and stem increment). Over all treatments and soil types, shoot increment was negatively correlated with starch (r = −0.497, p < 0.01) and total NSC (r = −0.524, p < 0.01), as well as nitrogen concentration (r = −0.277, p < 0.05) (Table 3). A significantly positive correlation, however, was found between shoot increment and the ratio of leaf soluble sugar to starch (r = 0.468, p < 0.01). Stem increment was negatively correlated only with leaf soluble sugar concentrations when all data were pooled (r = −0.270, p < 0.05). However, this trend was altered between different treatments, with a positive correlation occurring in the controls (r = 0.370, p < 0.05) and a negative correlation in the drought treatments (r = −0.256, p > 0.05).

## Discussion

### Drought effects

Drought resulted in increased leaf total NSC concentrations, but the magnitude differed between the two soil types (Table 1 and Fig. 1C). There are inconsistent results in respect to plant NSC responses to water deficit, such as accumulation/maintenance^21^,^22^,^23^,^24^,^56^ or depletions^57^,^58^,^59^. Recently, it has been proposed that plant NSC accumulation or depletion under water deficit depends on species-specific strategies^2^,^60^. In the present study, we aimed at testing how the leaf NSC accumulation representing the net effect of C gain *vs*. C use and export under drought may be related to growth (Fig. 1 and Table 3). As a result of the greater sensitivity of growth (turgor-driven cell expansion) compared to the sensitivity of photosynthesis to water deficit, moderate water stress is often associated with an increase in NSC and a ‘surplus’ of photosynthates which the plant is unable to use for the more drought-sensitive, turgor-driven cell growth^61^. Only recently, Hagedorn *et al*.^62^ provided this hypothesis as they observed an increase in NSC in roots and with a time lag also for leaves during drought onset. These authors, however, assumed that down-regulation of photosynthesis would counteract the accumulation of starch in leaves over the longer term. In the present study, this assumption could be confirmed, as there was rather a decrease in starch content on both soil types indicating reduced short-term storage. In contrast, there was a significant increase in sugar concentration on the acidic soil. Under water deficit, soluble sugar could aid in desiccation tolerance through osmotic adjustment and stabilization of membranes and proteins^57^. For beech, Ruehr *et al*.^63^ observed reduced export of recent assimilates out of the leaf into the phloem under drought supporting the hypothesis of osmotic adjustment. After stress relief, soluble sugar and total NSC concentration of previously drought-treated saplings recovered to the level of controls, which is in accordance with similar studies for *F. sylvatica* L. or other species^64^,^65^,^66^. Gallé & Feller^50^ found that net photosynthesis rate of *F. sylvatica* L. completely recovered within 4 weeks, meanwhile stomatal conductance remained permanently lower, leading to an increased ‘intrinsic water use efficiency’. We have now published a paper on photosynthesis in the same experiment showing full recovery within 2 to 3 weeks and “overshooting” photosynthesis after full recovery^51^.

Soil drought may increase soil solute concentrations, which might promote nutrient uptake since uptake is positively correlated to external solute concentrations. However, soil water availability could be most important due to the role of water as carrier in nutrient uptake and transport. A reduction of water availability may reduce nutrient diffusivity and mass flow^67^ and, in addition, a reduction of the root uptake capacity for nutrients has been observed^38^. In our study, the two observed nutrient elements (nitrogen and phosphorus) were only slightly or not at all affected by drought (Fig. 2). Our results are consistent with some studies^68^,^69^,^70^,^71^, but not with others^44^,^72^,^73^. For instance, by analyzing mineral nutrition of *F. sylvatica* L. seedlings from eleven provenances, Peuke & Rennenberg^74^ found that drought led to a reduction in leaf phosphorus concentration, but had no significant effects on leaf nitrogen. Based on a meta-analysis, He & Dijkstra^75^ suggested that, negative effects on plant nitrogen and phosphorus are alleviated with extended duration of drought and with drought-re-watering cycles. Over time, plants may adjust their growth, morphology, and physiochemical characteristics to acclimatize to water deficit. For example, by enhancing root growth and extension (increasing the root:shoot ratio) to absorb more water and nutrients from deeper soil layers^76^,^77^. Our results imply that nitrogen and phosphorus availability may not be limited for sapling growth in the present study, while a slight increase in leaf nitrogen content on acidic soil may reflect more soil nitrogen availability than on calcareous soil (Fig. 2).

### Soil effects

Soil properties (e.g. pH, texture, nutrient availability) could affect plant growth^53^,^78^ and it’s response to water deficit^3^,^42^,^79^. For instance, Kuster *et al*.^3^ reported that oaks produced more biomass on the acidic than on the calcareous soil in the absence of drought, while under water deficit, the relative growth reduction on the acidic soil was higher than on the calcareous soil. Also Thiel *et al*.^42^ found that drought negatively impacted growth of European beech, while the sandy substrate caused more growth reduction than the loamy substrate. However, our growth data showed that European beech saplings favored and were better adapted to the calcareous soil with a higher shoot increment, which is consistent with former studies for the same species^78^,^80^. In the present study, drought decreased the growth of plants on both soils to a comparable extent (Fig. 3). However, leaf soluble sugar and total NSC concentrations on acidic soil were higher than on calcareous soil under the control and drought treatment. This contrasting pattern between growth and leaf NSC on the two soil types implied that soil types associated with soil chemical and physical properties modify or even determine the availability of carbohydrates to plant growth. On the other hand, soil types may reduce the sink activity, which in turn may result in less NSC investment to growth and thus lead to decreasing growth rate but increasing NSC concentration. An increased leaf NSC concentration can in turn down-regulate photosynthesis^81^, and thus decreases the growth rate.

The present study revealed a negative correlation between shoot growth and leaf starch concentration, as well as a positive correlation between shoot growth and leaf soluble sugar:starch ratio in drought-stressed saplings. Similar results have been reported by Woodhams and Kozlowski^82^ more than a half-century ago. Leaf soluble sugars produced by photosynthesis export from the source leaves into the phloem, and are used directly for plant growth^83^. Hence, leaf sugars are a short-term pool and starch is a temporary storage induced due to either high carbon assimilation or low carbon export. On the other hand, drought may restrict the sink activity, and thus constrain the NSC export from leaves and NSC use, leading to decreased growth accompanied with leaf starch accumulation^84^. Up to now, there are only a few studies with herbaceous species showing that starch (or the ratio of soluble sugars to starch) is a major integrator in the regulation of plant growth^85^,^86^,^87^. Fast growing species for example, operate in a less conservative manner, diverting a slightly larger proportion of the newly assimilated carbon into soluble sugar export for growth and retaining less of starch to act as a short-term reserve or buffer against changes in environmental conditions^86^. Our results show the same for a woody, late successional plant species.

## Conclusions

The present study revealed that drought reduced shoot and stem growth of beech grown in acidic soil rather than in calcareous soil, indicating that soils with different physical and chemical properties can influence plants’ drought tolerance. We, therefore, suggest that soil type should be considered as an additional modifying parameter when examining the influences of stress (e.g. drought) on plants. Drought led, in general, to increased total NSC concentrations but decreased growth rate, which may imply a priority of carbon storage over growth for drought-stressed plants. The negative correlation between starch concentration and growth, as well as a positive correlation between soluble sugar:starch ratio and growth as revealed by this study, may contribute to a better understanding of growth regulation under drought conditions. We speculate that growth of drought-stressed trees depends upon a soluble sugar-starch relation rather than a higher level of the total NSC, which is needed to be further verified for other woody species.

## Materials and Methods

### Experimental design and treatments

The present study was conducted in the model ecosystem facility of the Swiss Federal Research Institute WSL (47°21′54″N, 8°27′5″E, 545 m a.s.l.), Birmensdorf, Switzerland. The facility consists of 16 ortho-hexagonal open top chambers (OTCs) of 3.5 m in height and 1.0 m in side length, each equipped with an automated irrigation system and a sliding roof closing automatically at the onset of rainfall. The experiment was designed as a split-plot experiment with whole-plot treatments control (8 OTCs) and drought/re-watering (8 OTCs)^53^. Drought development was followed by measurements of soil moisture in each lysimeter at 10 cm soil depth (5TM, Decagon, USA). Each OTC is split into two lysimeters with a plantable area of 3 m^2^ each. In each OTC, one of the two lysimeters was randomly selected to be filled with 100-cm-deep acidic (haplic Alisol) forest soil, and the other one with calcareous forest soil (sub-plot factor). The acidic and calcareous soils had a pH of 4.0 and 6.9, respectively, with different chemical composition but comparable soil texture (Table S1; see also Kuster *et al*.^3^; Arend *et al*.^51^). The most differing mineral elements were calcium with a 10 times higher availability in calcareous soil and manganese with a 13 times higher availability in acidic soil, respectively. In spring 2011, 24 saplings with ~20 cm in height from 12 *Fagus sylvatica* L. provenances (2 saplings each) were transplanted in each lysimeter (sub-sub-plot factor)^53^.

From November to April, the sliding roofs of the chambers were kept open to allow natural precipitation. By closing the sliding roofs from May to October, natural precipitation was excluded. The chambers were irrigated every second or third day with 50 l m^−2^ deionized water, enriched with nutrients to simulate the average composition of ambient rainfall (see also Kuster *et al*.^3^). During hot summer periods, the irrigation intensity and frequency was increased to counterbalance higher rates of evapotranspiration and hold the soil moisture at 10 cm soil depth above 20%. With this target value, soil moisture in deeper soil layers was above field capacity as indicated by a constant outflow of drainage water at the bottom of the lysimeters. In 2014, when the saplings had reached a height of up to 2 m, a severe, long-lasting summer drought was imposed in the 8 OTCs with drought treatment, by reducing irrigation from 22 May to 2 August, and the 8 controls were irrigated as described above. After the first saplings reached predawn water potentials below −2.0 MPa, the 8 drought OTCs were intensely re-watered for 1 day with 200 l m^−2^ and afterwards regularly irrigated as described above.

### Leaf sampling

To reduce the work and costs of chemical analysis, the present study used only 3 provenances originating from xeric, semi-xeric and mesic forest sites (Table S2). During 10:00am-03:00 pm, leaf material was collected from randomly chosen lysimeters under control (n = 5) or drought treatment (n = 5) (Table S2) on both soil types (n = 60), one day before re-watering (July 31^th^) and 50 days after re-watering (September 19^th^). In each lysimeter, a single sapling of the xeric, semi-xeric and mesic provenance was selected. For each individual, 5~8 healthy and fully developed leaves (depending on the leaf size) were harvested, and immediately frozen in liquid nitrogen, and stored at −80 °C. After being scanned for leaf area (Image J v1.48) to determine special leaf area (SLA, cm^2^/g DW), all samples were dried to a constant weight at 65 °C for 72 h. Finally, all samples were ground with a mixer mill MM400 (Retsch, Germany) for further analysis.

### Total soluble sugar and starch concentration

The powdered material (~50 mg) was put into a 10 ml centrifuge tube, where 5 ml of 80% ethanol was added. The mixture was incubated at 80 °C in a water bath shaker for 30 min, and then centrifuged at 10,000 rpm for 5 min. The pellets were extracted two more times with 80% ethanol. Supernatants were retained, combined and stored at −20 °C for soluble sugar determinations. The ethanol-insoluble pellet was used for starch extraction. Glucose was used as a standard. Soluble sugars were determined using the anthrone method^88^. The starch concentration was measured spectrophotometrically at 620 nm using anthrone reagent, and was calculated by multiplying glucose concentrations by the conversion factor of 0.9^89^. Concentration of soluble sugars and starch was expressed on a dry matter basis (% DW). Concentration of non-structural carbohydrates (NSC) was obtained by summing up the total soluble sugar and starch concentrations.

### Leaf nitrogen and phosphorus concentration

For determination of leaf nitrogen (N) and phosphorus (P) concentrations (mg/g DW), finely ground material (~50 mg) was firstly digested with H_2_SO_4_ and H_2_O_2_ for further analysis. Leaf nitrogen concentration was then measured using the Kjeldahl method (Kjeltec 2200, FOSS, Sweden), while leaf phosphorus concentration was determined with the molybdenum blue spectrophotometric procedure (6505 UV spectrophotometer, UK)^90^.

### Growth measurement

Annual shoot growth was determined on current-year leader shoots at the end of the growing season. Seasonal stem diameter increment was calculated from measurements of the stem diameter (10 cm above ground) at the start and end of the growing season.

### Statistical analysis

All statistical analyses were conducted by using R statistical software (RStudio version 0.98.953, http://www.rstudio.com/). Shapiro-Wilk and Bartlett’s tests were firstly used to test for normality and homogeneity of variances respectively, and all variables met the assumption for further variance analysis. Pre-analysis revealed no significant differences between provenances for all responsive variables (except for phosphorus). Therefore, we pooled the three provenances into one species. The linear mixed-effects models (R package-nlme) were used to determine the effects of three fixed factors, i.e. sampling period (July *vs*. September), treatment (control *vs*. drought-rewatering) and soil type (acidic *vs.* calcareous soil) on each response variable, provenance and growth chamber were considered as random factors. The response variables included leaf non-structural carbohydrate concentrations (soluble sugars, starch, total NSC), nutrient concentration (nitrogen, phosphorus) and growth increment (annual shoot and stem increment) on both soils. Significant differences in each response variable between the treatments within each soil type, as well as differences across soil types were compared by lsmeans (least squares means estimates, R package-lsmeans) and adjusted by the Tukey adjustment to identify pairwise differences (p < 0.05).

## Additional Information

**How to cite this article**: Liu, J.-F. *et al*. Effects of drought on leaf carbon source and growth of European beech are modulated by soil type. *Sci. Rep.* **7**, 42462; doi: 10.1038/srep42462 (2017).

**Publisher's note:** Springer Nature remains neutral with regard to jurisdictional claims in published maps and institutional affiliations.

## Supplementary Material

Supporting Information

## Figures and Tables

**Table 1:** Summary of three-way repeated measure ANOVA for the effects of treatment and soil type on leaf NSCs (soluble sugars, starch and NSC), nutrients (nitrogen and phophuros) in European beech saplings.

Source of variation	*df*	Soluble sugars		Starch		Total NSC		TN		TP	
		*F*	*P*	*F*	*P*	*F*	*P*	*F*	*P*	*F*	*P*
Within-subject											
Period	1	13.475	**0.001**	3.145	0.082	4.156	**0.046**	17.396	**<0.001**	42.404	**<0.001**
Period × Treatment	1	23.416	**<0.001**	26.858	**<0.001**	0.574	0.452	0.642	0.426	1.200	0.278
Period × Soil	1	12.731	**0.001**	61.298	**<0.001**	71.628	**<0.001**	1.993	0.164	11.770	**0.001**
Period × Treatment × Soil	1	10.249	**0.002**	0.054	0.818	7.129	**0.010**	0.765	0.385	0.503	0.481
Between-subject											
Treatment	1	17.057	**<0.001**	0.303	0.584	8.575	**0.005**	1.201	0.278	0.855	0.359
Soil	1	2.248	0.139	37.912	**<0.001**	30.614	**<0.001**	41.464	**<0.001**	21.458	**<0.001**
Treatment × Soil	1	1.923	0.171	2.526	0.118	0.001	0.997	4.280	**0.043**	0.294	0.590

**Table 2:** Summary of two-way ANOVA for the effects of treatment and soil type on shoot- (cm yr^−1^), stem- growth (mm yr^−1^) and SLA (cm^2^/g) of *Fagus sylvatica* L. saplings.

Source of variation	*df*	Shoot increment (H)		Stem increment (D)		SLA	
		*F*	*P*	*F*	*P*	*F*	*P*
Treatment	1	3.24	0.093	19.91	**<0.001**	0.02	0.833
Soil	1	27.52	**<0.001**	2.13	0.152	5.64	**0.028**
Treatment × Soil	1	0.00	0.980	0.02	0.882	1.27	0.274

**Table 3:** Correlation analysis between growth parameters (H: shoot increment (cm); D: stem increment (mm)), carbon (soluble sugars, starch and total NSC) and nutrients (nitrogen and phosphorus), SLA (cm^2^/g) measured in July (before re-watering) for *Fagus sylvatica* L. saplings grown on different soils (acidic *vs*. calcareous) under different treatments (control *vs*. drought).

			Control (n = 30)						Drought (n = 30)					
	Total		Sub-total		Acidic soil		Calcareous soil		Sub-total		Acidic soil		Calcareous soil	
	H	D	H	D	H	D	H	D	H	D	H	D	H	D
Soluble sugars	0.243	−0.270*	**−**0.008	0.370*	**−**0.003	0.447	0.056	0.242	**−**0.293	**−**0.256	**−**0.037	**−**0.210	0.295	**−**0.097
Starch	−0.497**	0.061	−0.588**	**−**0.150	**−**0.225	**−**0.165	**−**0.413	0.408	−0.587**	−0.371*	**−**0.376	**−**0.406	**−**0.249	**−**0.338
NSC	−0.524**	0.240	−0.508**	0.093	**−**0.170	0.259	**−**0.193	0.472	−0.505**	−0.364*	**−**0.307	**−**0.462	0.245	**−**0.186
TN	−0.277*	0.229	−0.404*	**−**0.319	**−**0.462	**−**0.300	**−**0.305	**−**0.474	**−**0.120	**−**0.055	0.354	0.092	0.510	0.127
TP	0.099	0.003	**−**0.129	**−**0.062	**−**0.187	**−**0.408	**−**0.009	0.356	**−**0.070	0.061	0.385	0.063	**−**0.123	0.145
Soluble sugars:Starch	0.468**	0.017	0.619**	0.148	0.292	0.422	0.401	**−**0.235	0.621**	0.338	0.298	0.338	0.332	0.238
SLA	**−**0.109	**−**0.151	**−**0.084	**−**0.218	0.530*	**−**0.172	**−**0.345	**−**0.155	**−**0.135	**−**0.097	0.371	**−**0.071	**−**0.356	**−**0.051

Numbers in bold indicate statistical significance at the 5% level, where *p < 0.05; **p < 0.01.
