# Supplementary material for: Effects of drought on leaf carbon source and growth of European beech are modulated by soil type
Source: Sci Rep. 2017 Feb 14;7:42462. doi: 10.1038/srep42462 (PMC5307967; doi:10.1038/srep42462)
Supplement: Supporting Information [file srep42462-s1.pdf]

Effects of drought on leaf carbon source and growth of European beech are modulated by soil type

Jian-Feng Liu[a,b,#], Matthias Arend[b,e, #], Wen-Juan Yang[a], Marcus Schaub[b], Yan-Yan Ni[a], Arthur Gessler[b,c], Ze-Ping Jiang[a], Andreas Rigling[b], Mai-He Li[b,d, *]

Table S1. Physical and chemical characteristics of the acidic and calcareous soil type (data from Kuster *et al*., 2013 with permission of the publisher Wiley-Blackwell). CEC: cation exchange capacity; the exchangeable cations (subscript: exch.) were measured in 0.1 M $BaCl_2$ extracts.

Table S2. Beech provenances transplanted to the WSL Model Ecosystem Facility MODOEK and environmental characteristics of the provenance sites. Climate data (annual mean temperature and annual sums of precipitation) taken from nearby METEO SWISS stations (distances ≤ 10 km).

Table S1. Physical and chemical characteristics of the acidic and calcareous soil type (data from Kuster *et al*., 2013, with permission of the publisher Wiley-Blackwell). CEC: cation exchange capacity; the exchangeable cations (subscript: exch.) were measured in 0.1 M $BaCl_2$ extracts.

	acidic soil	calcareous soil
Texture (% sand, silt, clay)	87, 8, 5	71, 18, 12
pH (0.01 M $CaCl_2$)	4.0	6.9
N_{tot} (%)	0.03	0.05
P_{tot} (mg kg^{-1})	469	358
$Ca_{exch.}$ (mg kg^{-1})	142	1629
$Mg_{exch.}$ (mg kg^{-1})	9.5	21.9
$K_{exch.}$ (mg kg^{-1})	19.0	24.7
$Mn_{exch.}$ (mg kg^{-1})	18.6	1.4
CEC (mmol kg^{-1})	24.1	84.7
Base saturation (%)	36.7	99.4

Table S2. Beech provenances transplanted to the WSL Model Ecosystem Facility MODOEK and environmental characteristics of the provenance sites. Climate data (annual mean temperature and annual sums of precipitation) taken from nearby METEO SWISS stations (distances ⩽ 10 km).

Provenance	Geographic location	Elevation [m a.s.l.]	Annual mean temperature [℃]	Annual precipitation [mm]
Saxon	46°08’N, 7°11’E	700-800	9.2	542
Martigny	46°06’N, 7°06’E	500-700	9.2	843
Collombey	46°16’N, 6°56’E	550-650	8.9	1055
